# Lateral-to-Medial Femoral Condyle Length Ratio on Standard AP Knee Radiographs as a Predictor of Patellar Chondromalacia

**DOI:** 10.3390/jcm15124535

**Published:** 2026-06-11

**Authors:** Bedirhan Albayrak, Furkan Tabur, Furkan Erdoğan, Ferhat Say

**Affiliations:** 1 Department of Orthopaedics and Traumatology, Samsun City Hospital, Samsun 55080, Turkey; 2 Department of Orthopaedics and Traumatology, Ondokuz Mayıs University, Samsun 55100, Turkey; furkantabur.185@hotmail.com (F.T.); erdogan27@yahoo.com (F.E.); ferhatsay@gmail.com (F.S.)

**Keywords:** patellofemoral joint, chondromalacia patellae, patellofemoral pain syndrome

## Abstract

**Background/Objectives**: Patellar chondromalacia (PC) is a clinical condition characterized by early cartilage degeneration in the patellofemoral joint. This study aimed to investigate the relationship between the lateral-to-medial femoral condyle length ratio (LFCL/MFCL) measured on standard anteroposterior (AP) knee radiographs and the presence of PC. **Methods**: A retrospective analysis was conducted on patients who presented with anterior knee pain between 2020 and 2024. PC was diagnosed using magnetic resonance imaging (MRI). The LFCL/MFCL ratio was measured on plain radiographs. Additional morphological condylar parameters were evaluated on MRI. Symptom severity was assessed using the Kujala score. Statistical analyses included *t*-tests, correlation analysis, logistic regression, and receiver operating characteristic (ROC) curve analysis. **Results**: A total of 100 patients (50 with PC, 50 controls) were included. The LFCL/MFCL ratio was significantly higher in the PC group compared to controls (1.24 ± 0.19 vs. 1.08 ± 0.15, *p* = 0.002). A negative correlation was found between the LFCL/MFCL ratio and Kujala score (r = −0.322, *p* = 0.029). Other MRI-based parameters did not show statistically significant differences. In logistic regression, the LFCL/MFCL ratio was identified as an independent predictor of PC (*p* = 0.01). ROC analysis yielded an AUC of 0.743 (95% CI: 0.643–0.842). **Conclusions**: The LFCL/MFCL ratio, which can be easily measured on plain radiographs, may serve as a simple, cost-effective, and reproducible parameter to aid in the diagnosis of patellar chondromalacia. Further prospective studies with larger sample sizes are needed to validate this finding.

## 1. Introduction

The physiological function of the patellofemoral joint depends on the harmonious anatomical alignment between the patella and the femoral trochlear groove. Developmental or acquired alterations in trochlear morphology have been associated with clinical conditions such as anterior knee pain, patellar chondromalacia (PC), and advanced-stage osteoarthritis [1]. Cases of softening of the patellar cartilage have been reported in almost half of healthy individuals over the age of 20 and in virtually every individual over the age of 50 [2]. The primary causes include trauma to the knee region, repetitive microtrauma, sports injuries, osteochondritis dissecans resulting from vascular disorders, and inflammatory diseases [3].

Trochlear dysplasia has been identified as the underlying anatomical abnormality in approximately 96% of cases of patellofemoral instability [4]. Trochlear dysplasia associated with patellar instability can alter patellofemoral biomechanics, even though it does not cause instability in milder cases. Various hypotheses regarding the etiopathogenesis of trochlear dysplasia include medial femoral condyle hypoplasia, lateral condyle hypertrophy, or a combination of both, as well as morphological changes in the distal femur associated with increased femoral anteversion [5,6,7].

Current imaging modalities, including magnetic resonance imaging (MRI) and three-dimensional computed tomography (3D-CT), allow for detailed assessment of trochlear morphology and patellofemoral congruence using parameters such as trochlear depth (TD), sulcus angle (SA), lateral patellar tilt angle (LPTA), and the Insall–Salvati index (ISI). Recent studies have emphasized that comparative measurements between medial and lateral femoral condyle morphology may be associated with PC. In this context, evaluating diameters and lengths of the femoral condyles, as well as their ratios, is gaining increasing importance in identifying patellofemoral pathologies [5,8,9,10].

These measurements require advanced imaging modalities and are not always readily accessible in routine clinical practice. Although various morphometric indices have been described in the literature, a simple, widely accepted radiographic parameter that can be easily obtained from standard knee radiographs remains lacking. Therefore, there is a need for practical, accessible markers to assist in the early prediction of patellar chondromalacia. In this context, although standard anteroposterior knee radiographs with the patella positioned centrally are considered optimal for evaluation, underlying femoral torsional variations may still influence the apparent morphology of the distal femur [11]. Given that femoral torsion reflects the rotational relationship between the femoral neck and distal condyles, such variations can lead to projectional differences on plain radiographs, potentially causing the medial femoral condyle to appear relatively smaller due to its altered orientation relative to the X-ray beam. In this context, the lateral-to-medial femoral condyle length ratio (LFCL/MFCL) could represent an indirect radiographic manifestation of mild femoral torsional variations.

PC, an early stage of patellofemoral joint degeneration, can be managed with surgical correction or lifestyle modifications when diagnosed in a timely manner. The present study aimed to evaluate the relationship between the LFCL/MFCL ratio measured on standard anteroposterior knee radiographs and PC, and to investigate the potential of this ratio as a radiographic predictor.

## 2. Materials and Methods

### 2.1. Study Design

Single-center retrospective cross-sectional study.

### 2.2. Study Population and Data Collection

The study was conducted by reviewing the reviewing demographic characteristics, clinical records, and imaging data of patients who presented to our orthopedic outpatient clinic with complaints of anterior knee pain between 2020 and 2024. The study was approved by the Ondokuz Mayıs University Clinical Research Ethics Committee (approval number: 2024/214).

Eligible patients were divided into two groups: symptomatic individuals with isolated patellar chondromalacia (PC) confirmed on knee MRI (case group) and individuals with nonspecific anterior knee pain and no detectable pathology on MRI (control group). The inclusion criteria were age between 17 and 55 years, anterior knee pain as the primary complaint, and MRI-confirmed PC for the case group. To ensure standardization of isolated PC cases, patients with any concomitant intra-articular pathology on MRI, including osteochondritis dissecans, meniscal lesions, ligament injuries, or other associated abnormalities, were excluded. Additional exclusion criteria included rotational deformities, history of knee surgery, fractures around the femur or tibia, systemic inflammatory diseases (e.g., rheumatoid arthritis), calcium pyrophosphate disease detected by synovial fluid analysis, and advanced-stage tibiofemoral or grade 3–4 patellofemoral osteoarthritis on MRI. Tibiofemoral osteoarthritis was evaluated according to the Kellgren–Lawrence classification, while patellofemoral osteoarthritis was assessed using the Iwano classification.

The severity of patellofemoral symptoms was assessed using the Kujala anterior knee pain score [12]. To ensure homogeneity of the study population, only patients with a Kujala score ≤60 and MRI-confirmed isolated PC were included in the case group. A total of 850 knee MRI examinations were screened; 220 patients were excluded due to age criteria, and 563 patients were excluded due to the presence of additional MRI-detected pathologies other than PC. Patients with MRI-confirmed isolated PC were subsequently invited for outpatient evaluation. Among these, 17 patients with a Kujala score >60 at presentation or with symptom resolution following treatment were excluded to minimize clinical heterogeneity. As a result, 50 patients (27 females, 23 males) with isolated PC meeting all inclusion criteria were included in the case group. This study was designed and reported in accordance with the STROBE guidelines.

**Figure 1:**
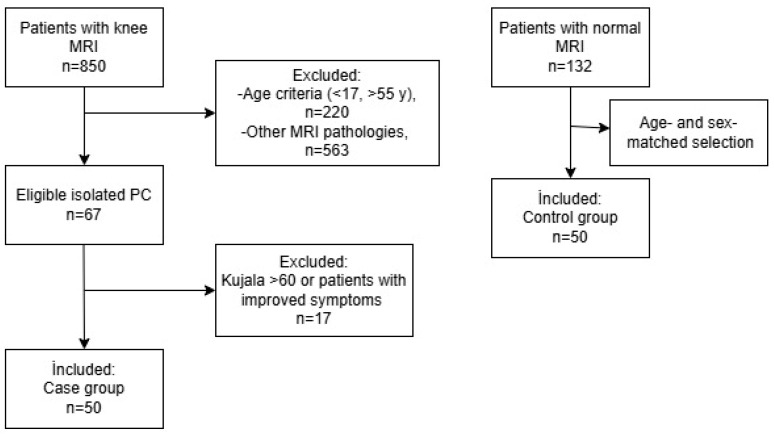
Flowchart of patient selection. From 850 knee MRI examinations, 67 patients with isolated patellar chondromalacia were identified after exclusions; 50 met clinical criteria and were included. The control group comprised 50 age- and sex-matched individuals selected from 132 patients with normal MRI findings.

The control group consisted of individuals who presented to the outpatient clinic during the same period with nonspecific, mild anterior knee pain and no structural abnormalities on MRI. These individuals were matched to the case group by age and sex and were selected using a simple random sampling method from a pool of 132 eligible candidates. Ultimately, 50 individuals (24 females, 26 males) were included in the control group (Figure 1).

Radiographic measurements were performed by an independent observer who was not involved in the clinical evaluation. Clinical assessments, including Kujala scores, were conducted by a separate investigator. The radiographic evaluator was blinded to group allocation (case vs. control) and to all clinical data.

### 2.3. Radiographic and MRI Measurements

**Figure 2:**
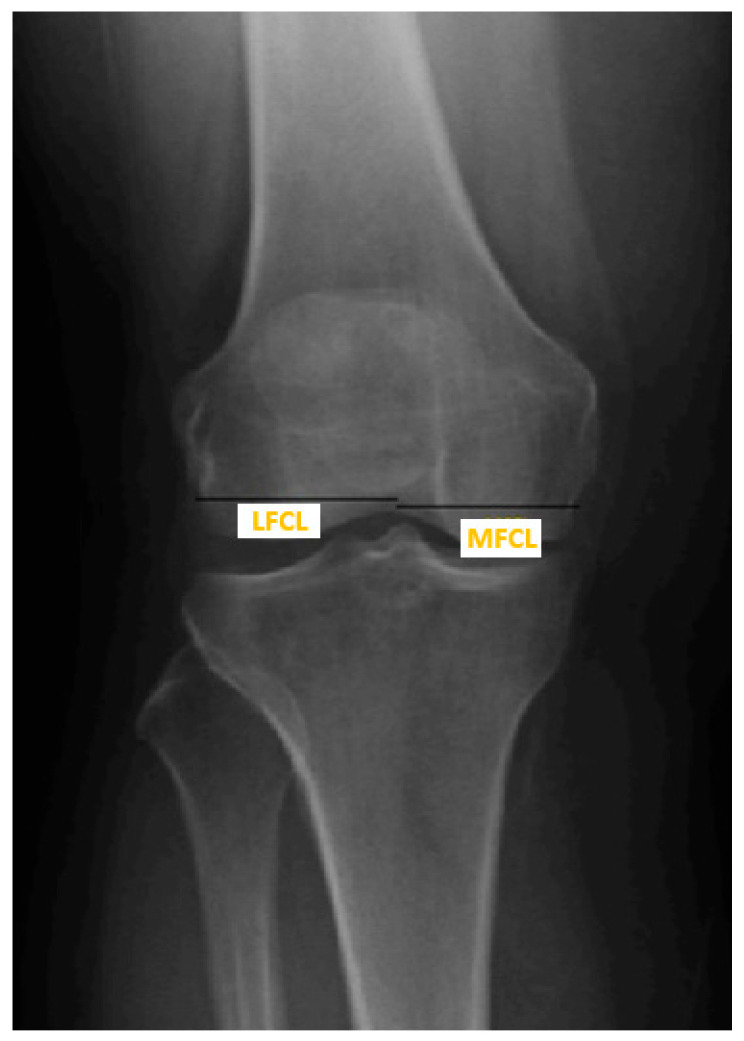
The lateral and medial femoral condyle lengths were measured on the plain radiograph.

On standard anteroposterior (AP) knee radiographs, measurements of the medial femoral condyle (MFCL) and lateral femoral condyle (LFCL) widths were performed using images in which the patella was centered. On the anteroposterior radiograph, measurements were performed by drawing lines parallel to the ground from the deepest point of the trochlear notch to the medial and lateral epicondyles. All measurements were calculated using values obtained from full-length lower extremity radiographs (Figure 2). The LFCL/MFCL ratio was calculated based on the distance from the deepest point of the trochlear groove to the most prominent point of each condyle.

**Figure 3:**
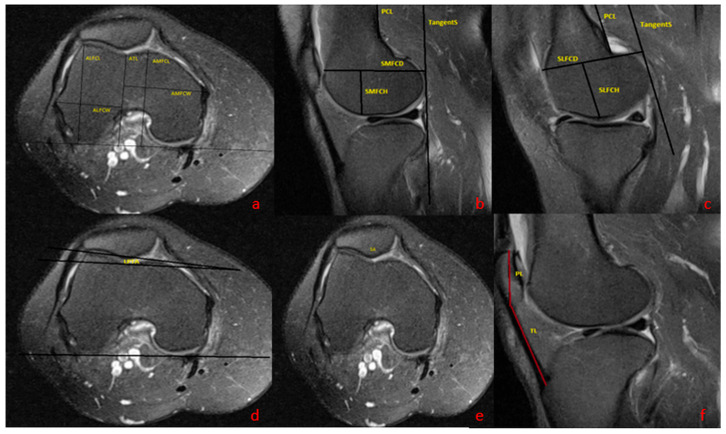
(**a**). Length measurements on axial MRI, (**b**). Medial condyle parameters on sagittal MRI, (**c**). Lateral condyle parameters on sagittal MRI, (**d**). LPTA measurement, (**e**). Sulcus angle measurement, (**f**). Insall-Salvati ratio measurement.

MRI examinations were performed using 1.5 T scanners, and all parameters were evaluated on axial and sagittal planes using T2-weighted sequences with 3 mm slice thickness. On MRI, axial plane measurements included: anterior medial femoral condyle width (AMFCW) and length (AMFCL), anterior lateral femoral condyle width (ALFCW) and length (ALFCL), anterior trochlear length (ATL), sulcus angle (SA), lateral patellar tilt angle (LPTA), and trochlear depth (TD) (Figure 3a,d,e). These measurements were obtained at the axial slice where the femoral condyles appeared widest. Using a tangent line (tangentA) touching the posterior edges of the condyles as reference, AMFCL, ALFCL, and ATL were measured. Perpendicular lines drawn from the ATL line to the widest points of the medial and lateral condyles were used to determine AMFCW and ALFCW (Figure 3a).

In the sagittal plane, measurements were taken at the slice where the condyles appeared largest. These included sagittal medial femoral condyle depth (SMFCD) and height (SMFCH), sagittal lateral femoral condyle depth (SLFCD) and height (SLFCH), and the tendon length (TL)/patellar length (PL) ratio for calculating the Insall–Salvati index (ISI) (Figure 3b,c,f). The reference line (tangentS), parallel to the posterior condylar line (PCL), was used to measure SMFCD and SLFCD. The vertical distance from this line to the apex of the condyle was recorded as SMFCH and SLFCH, respectively (Figure 3b,c).

All measurements were expressed as ratios to minimize the effect of inter-individual anatomical variation. These included the axial width ratio (ALFCW/AMFCW), axial length ratio (ALFCL/AMFCL), sagittal depth ratio (SLFCD/SMFCD), sagittal height ratio (SLFCH/SMFCH), and the condylar width ratio (LFCL/MFCL) obtained from direct radiographs. All measurements were performed twice, two weeks apart, by the same orthopedic surgeon with seven years of experience. Intraobserver reliability was evaluated by repeating all measurements twice at a two-week interval by the same observer. A strong correlation was observed between the repeated measurements (r = 0.91, *p* < 0.001), and the first set of measurements was used for further statistical analysis.

The collected data were statistically compared between the case and control groups. Additionally, statistical analyses were performed to evaluate the relationship between imaging findings and Kujala scores.

### 2.4. Statistical Analyses

The data were analyzed using SPSS software version 22.0 (IBM Corp., Armonk, NY, USA). The distribution of continuous variables was assessed using the Kolmogorov–Smirnov test. For variables with normal distribution, the independent samples *t*-test was used, whereas the Mann–Whitney U test was applied for non-normally distributed variables. The Chi-square test was used for categorical variables. Logistic regression analysis was performed to identify independent predictors of patellar chondromalacia, and receiver operating characteristic (ROC) analysis was used to evaluate diagnostic performance. Intraobserver reliability between repeated measurements performed by the same observer was assessed using the Pearson correlation coefficient. A *p*-value of < 0.05 was considered statistically significant.

## 3. Results

A total of 100 individuals were included in the study. The PC group consisted of 50 patients (27 females, 23 males; mean age: 44 ± 10.2 years) diagnosed with PC on MRI and a Kujala anterior knee pain score ≤60. The control group included 50 individuals (24 females, 26 males; mean age: 39 ± 8.3 years), selected from 132 candidates presenting with normal MRI findings, matched for age and sex.

Among all radiographic and MRI-based morphological parameters, only the LFCL/MFCL ratio demonstrated a statistically significant difference between the case and control groups (*p* = 0.002). No significant differences were observed for the other parameters, including ALFCL/AMFCL, ALFCW/AMFCW, SLFCD/SMFCD, and SLFCH/SMFCH (*p* > 0.05) (Table 1).

In this study, patients with patellar chondromalacia had significantly lower lateral patellar tilt angle (LPTA) and trochlear depth (TD) values, while other parameters such as sulcus angle (SA) and Insall–Salvati index (ISI) showed no significant differences (Table 2).

Correlation analysis revealed a weak but significant negative correlation between the LFCL/MFCL ratio and Kujala score (r = −0.322, *p* = 0.029), suggesting that increased lateral femoral condyle length may be associated with poorer functional outcomes and that structural morphological changes may be linked to clinical symptoms. Additionally, a weak-to-moderate negative correlation was observed between trochlear depth (TD) and the ALFCL/AMFCL ratio (r = −0.365, *p* = 0.013), indicating a possible association between greater lateral femoral condyle length and trochlear shallowness.

Variables included in the logistic regression model were selected based on univariate analysis results and their clinical relevance and comprised the LFCL/MFCL ratio, LPTA, and TD. In the logistic regression analysis, only the LFCL/MFCL ratio emerged as a statistically significant independent predictor of patellar chondromalacia (*p* = 0.010). Other MRI-based parameters, including LPTA, TD, and ISI, were not identified as significant predictors. The model’s overall accuracy was 76.1%.

ROC analysis demonstrated that the LFCL/MFCL ratio had an area under the curve (AUC) of 0.743 (95% CI: 0.581–0.883) for predicting patellar chondromalacia, indicating a moderate discriminative ability. The optimal cut-off value was determined as 1.29 based on the Youden index. At this threshold, the sensitivity was 52.4%, the specificity was 92.0%, the positive predictive value (PPV) was 84.6%, and the negative predictive value (NPV) was 69.7%.

## 4. Discussion

In the present study, we investigated the relationship between the LFCL/MFCL ratio measured on standard anteroposterior knee radiographs and patellar chondromalacia (PC), and evaluated its potential role as a simple radiographic predictor. Our findings demonstrated that the LFCL/MFCL ratio was significantly higher in patients with PC and was the only independent predictor identified in multivariate analysis. In addition, a negative correlation between the LFCL/MFCL ratio and Kujala score suggests that this morphological parameter may be associated not only with structural alterations but also with clinical symptom severity. These results support our initial hypothesis that a simple radiographic ratio may reflect underlying patellofemoral morphological changes and could be useful in the early identification of PC.

Trochlear dysplasia and patellar chondromalacia (PC) are pathologies that disrupt the biomechanics of the patellofemoral joint and predispose to progressive cartilage damage and osteoarthritis [13]. In MRI studies conducted on individuals with PC, significant differences have been reported in parameters such as trochlear sulcus angle (SA), trochlear depth (TD), lateral patellar tilt angle (LPTA), lateral trochlear inclination (LTI), and Insall–Salvati index (ISI) [6,13,14]. Particularly, a decrease in TD and LPTA and an increase in SA have been reported to be significantly associated with PC. In our study, significant differences were also found in TD and LPTA values, while SA and ISI did not show statistical significance. The absence of statistical significance for some parameters may be attributed to the stage of disease at the time of evaluation. While certain morphometric parameters have been shown to be significant in advanced patellofemoral osteoarthritis [15], these changes may not yet be fully established in earlier-stage patellar chondromalacia, as represented in our study population.

Previous studies by Tabary and Lu have highlighted the predictive value of MRI-based parameters such as LPTA, TD, and ISI [5,16]. However, in our study, these parameters were not identified as significant predictors. These differences may be related to sample size, patient selection, measurement technique, or variability in symptom severity. In contrast, the LFCL/MFCL ratio is a rarely addressed and obtained from standard radiographic measurements parameter in the literature. This study is the first to systematically associate it with PC. Given its simplicity and accessibility, we believe it could serve as a supportive tool in risk stratification before advanced imaging in certain clinical scenarios.

PC is typically characterized by anterior knee pain, often triggered by activities such as squatting or climbing stairs. The Kujala score, used to objectively assess the functional impact of such symptoms, is a valuable measure for correlating structural changes with clinical manifestations and has been validated in Turkish [12]. The observed negative correlation between the LFCL/MFCL ratio and the Kujala score suggests that this radiographic parameter may be meaningful not only structurally but also symptomatically. While morphometric MRI-based analyses are widely reported for diagnosing PC, studies assessing functional metrics like the Kujala score remain limited. In this respect, our study makes a unique contribution by comprehensively examining the relationships among symptoms, function, and morphology.

It is known that femoral anteversion is higher in patients with dysplasia and that this increase is often associated with torsional changes in distal femur morphology [7,17,18]. Liu et al. associated the presence of a larger posteromedial and a smaller posterolateral femoral condyle with dysplasia [18]; Yang and Liebensteiner reported that increased distal femoral torsion correlates with dysplasia and is accompanied by notable asymmetries in femoral condyle morphology [9,17]. Although these anteversion parameters were not directly evaluated in our study due to the lack of routine CT imaging in this patient group, it should be considered that even in standard anteroposterior knee radiographs obtained with the patella positioned centrally, underlying femoral torsional variations may influence the apparent morphology of the distal femur. Given that femoral torsion reflects the rotational relationship between the femoral neck and distal condyles, such variations may lead to projectional differences on plain radiographs, potentially causing the medial femoral condyle to appear relatively smaller due to its altered orientation relative to the X-ray beam. In this context, an increased LFCL/MFCL ratio may represent a radiographic reflection of these morphological alterations and underlying torsional variations.

The literature also reports a positive correlation between medial tibial slope and femoral offset, particularly in individuals with Dejour type 4 dysplasia, and that dimensional enlargement of the lateral femoral condyle may indicate medial condyle hypoplasia [10]. In line with our findings, Jacob et al. demonstrated a clear association between trochlear dysplasia and medial femoral condyle hypoplasia, reporting significantly increased lateral-to-medial condylar ratios in patients with established dysplasia [19]. In contrast to that study, our cohort did not include patients with clearly defined or advanced trochlear dysplasia. Therefore, the increased LFCL/MFCL ratio observed in our study may reflect more subtle or early morphological variations rather than overt dysplastic changes. This finding suggests that even in the absence of pronounced trochlear dysplasia, mild femoral condylar asymmetry—potentially related to underlying torsional or morphological alterations—may still be detectable on standard radiographs. While such measurements were not included in our study, the increased LFCL/MFCL ratio may reflect morphological asymmetry between the femoral condyles. Supporting this hypothesis with advanced CT or 3D modeling may be recommended for future studies [20].

In imaging-based morphometric analyses, the choice of imaging plane and reference section can directly affect outcomes. In axial or sagittal plane measurements, technical differences such as selecting the widest femoral condyle slice or the most prominent level of the anterior femoral cortex may result in significant variation in parameter values [7,8]. Therefore, standardizing measurement protocols would improve diagnostic accuracy and enhance comparability between studies.

Milanovic et al., in their review focusing on adolescent athletes, emphasized that repetitive microtrauma, overuse, and patellofemoral biomechanical abnormalities play significant roles in the development of cartilage damage [21]. However, they also highlighted that patellofemoral malalignment, instability, and trochlear morphological variations may predispose young individuals to anterior knee pain and early cartilage degeneration. Although plain radiographs may be limited in detecting early chondral changes, early recognition of underlying morphological abnormalities may still provide clinically relevant information, particularly when combined with appropriate preventive strategies such as activity modification, quadriceps strengthening exercises, and correction of biomechanical imbalances [22]. Initial management in young individuals identified as being at risk may also include flexibility exercises and rehabilitation programs targeting quadriceps, hip, and core musculature to improve patellar tracking and lower-extremity biomechanics. In patients with persistent symptoms despite conservative treatment, adjunctive treatment modalities such as injectable therapies, including platelet-rich plasma (PRP) in selected cases without major anatomical abnormalities, may also be considered [23]. In cases of persistent instability or significant structural abnormalities despite non-operative management, individualized surgical options may be evaluated according to the underlying pathology. These may include stabilization and realignment procedures such as medial patellofemoral ligament reconstruction or tibial tubercle osteotomy, as well as cartilage restoration procedures including microfracture, autologous matrix-induced chondrogenesis (AMIC), and autologous chondrocyte implantation (ACI/mACI) in selected patients with focal chondral lesions [24]. These interventions may potentially help slow disease progression and improve functional outcomes in at-risk patients.

Limitations of this study include its retrospective design and the inability to evaluate complementary morphological parameters such as femoral torsion and tibial slope. Positional variation during imaging may have affected measurement accuracy. Additionally, due to the broad age range of the sample group, the effects of age-related degenerative changes may not have been fully eliminated.

## 5. Conclusions

The LFCL/MFCL ratio measured on standard plain radiographs may serve as a simple and accessible parameter associated with patellar chondromalacia. While MRI remains the gold standard due to its ability to directly visualize cartilage damage, the LFCL/MFCL ratio may reflect underlying morphological changes of the patellofemoral joint and provide supportive information during initial clinical evaluation prior to advanced imaging. However, this ratio should not be considered a substitute for MRI, but rather a preliminary screening clue, particularly in resource-limited settings. Further prospective, multicenter studies are needed to better define its clinical applicability and underlying mechanisms.

## Figures and Tables

**Table 1:** Length measurement parameters according to the presence of patellar chondromalacia.

	Patellar Chondromalacia		
	Yes	No	*p* Value
ALFCL/AMFCL	1.02 ± 0.04	1.02 ± 0.04	0.967
ALFCW/AMFCW	1.21 ± 0.09	1.16 ± 0.08	0.604
SLFCD/SMFCD	1.07 ± 0.05	1.07 ± 0.04	0.940
SLFCH/SMFCH	0.99 ± 0.11	1.01 ± 0.1	0.651
LFCL/MFCL	1.24 ± 0.19	1.08 ± 0.15	0.002 *

* *p* value < 0.05.

**Table 2:** SA, LPTA, ISI, and TD parameters based on patellar chondromalacia.

	Patellar Chondromalacia		
	Yes	No	*p* Value
SA	140.6 ± 9.7	140.6 ± 6.2	0.484
LPTA	10.3 ± 4.1	14.6 ± 5.7	0.004 *
ISI	1.23 ± 0.2	1.25 ± 0.18	0.874
TD	5.09 ± 1.6	5.39 ± 1.16	0.047 *

* *p* value < 0.05.

## Data Availability

The data are not publicly available due to restrictions imposed by the institutional ethics committee, which did not approve data sharing with external centers.

## Abbreviations

The following abbreviations are used in this manuscript:

PC	Patellar Chondromalacia
AP	Anteroposterior
MRI	Magnetic Resonance Imaging
LFCL	Lateral Femoral Condyle Length
MFCL	Medial Femoral Condyle Length
LFCL/MFCL	Lateral-to-Medial Femoral Condyle Length Ratio
AMFCW	Axial Medial Femoral Condyle Width
ALFCW	Axial Lateral Femoral Condyle Width
AMFCL	Axial Medial Femoral Condyle Length
ALFCL	Axial Lateral Femoral Condyle Length
ATL	Anterior Trochlear Length
SA	Sulcus Angle
LPTA	Lateral Patellar Tilt Angle
TD	Trochlear Depth
SMFCD	Sagittal Medial Femoral Condyle Depth
SLFCD	Sagittal Lateral Femoral Condyle Depth
SMFCH	Sagittal Medial Femoral Condyle Height
SLFCH	Sagittal Lateral Femoral Condyle Height
ISI	Insall–Salvati Index
ROC	Receiver Operating Characteristic
AUC	Area Under the Curve
CI	Confidence Interval
